# Molecular characterisation of the caprine (*Capra hircus*) lymphocyte function-associated antigen-1 alpha subunit-encoding cDNA

**DOI:** 10.1186/1746-6148-1-4

**Published:** 2005-10-10

**Authors:** Thomas Fett, Laurent LM Zecchinon, Etienne A Baise, Daniel JM Desmecht

**Affiliations:** 1 Pathology Department, Faculty of Veterinary Medicine, University of Liege, 4000 Liege, Belgium

## Abstract

**Background:**

Lymphocyte function-associated antigen-1 (LFA-1, CD11a/CD18, alpha L beta 2) is required for many cellular adhesive interactions during the immune response.

**Results:**

The *Capra hircus *CD11a-encoding cDNA was sequenced and compared with its human, murine, rat, bovine and ovine counterparts. Despite some focal differences, it shares all the main characteristics of its known mammalian homologues.

**Conclusion:**

Therefore, along with the caprine CD18-encoding cDNA, which has been available for a few months, the sequence data revealed here will allow the *Capra hircus *LFA-1 expression *in vitro *as a tool to explore the specificities of inflammation in the caprine species.

## Background

Lymphocyte function-associated antigen-1 (LFA-1, α_L_β_2_, CD11a/CD18) is a member of the β_2_-integrin subfamily of cell surface receptors. Integrins consist of a 120 to 180 kDa α subunit (CD11a in this case) and a 90 to 110 kDa β subunit that are noncovalently associated single-pass transmembrane proteins [1]. The bulk of each integrin subunit is extracellular, where it typically functions as a receptor for extracellular matrix molecules or as a counterreceptor for surface proteins of apposed cells [2]. The heterodimer CD11a/CD18 is expressed on all leukocytes and mediates high affinity adhesion to a variety of cell types that express one or more of the β_2_-integrins ligands, intercellular adhesion molecules (ICAM-1 to -5) [3-5]. The adhesion process mediated is a critical step of a wide range of immunological activities, including cytolysis of target cells, cross-interaction and cross-stimulation between lymphocytes, phagocytosis of complement-coated targets, neutrophils clearance from inflamed tissue, and the regulation of leukocyte traffic between the bloodstream and tissues [6-9]. As the relevance of the goat model for studying leukocyte traffic, diapedesis and pathologic tissue infiltration is well established in such important areas as mastitis [10-13] or lentivirus-associated diseases [14-16], increasing our knowledge about caprine β_2 _integrins is of great importance to offer new possibilities for research and to provide additional insights into those fields. Along with the caprine CD18-encoding cDNA, which is available for a few months [17], the sequence data provided here will allow the *Capra hircus *β_2_-integrin CD11a/CD18 expression *in vitro *as a tool to examine the specificities of inflammation in the caprine species.

## Methods

### RNA isolation

Total RNA from phorbol myristate acetate (PMA)-stimulated (25 ng/ml for 15 min) caprine (*Boer *breed) peripheral blood mononuclear cells (PBMC) was extracted with TRIzol (Invitrogen, USA) as described by the manufacturer. The PBMCs were obtained by density gradient centrifugation with Ficoll-Paque Plus (Amersham, USA) and maintained in RPMI 1640 supplemented with 10% foetal bovine serum (Gibco BRL, USA), penicillin (100 U/ml) and streptomycin (100 μg/ml) at 37°C in a 5% CO_2 _atmosphere.

### Amplification of cDNA ends

We used SMART RACE technology (Clontech Laboratories Inc., USA) to obtain caprine CD11a (CaCD11a) 5'- and 3'-ends and RT-PCR to amplify full-length CaCD11a CDS. For first-strand cDNA synthesis, and according to the sequence of bovine CD11a available [GenBank: AY267467], gene-specific primers were designed which were expected to give non overlapping ~1 kb RACE products: a sense primer for the 3'-RACE PCR : 5'-TGCAATGTRAGCTCTCCCATCTTC-3' (corresponding to nt 2572 to nt 2595) and an antisense primer for the 5'-RACE PCR : 5'-CCGGCCTCCTCTCTGCTCCCCATAG-3' (nt 1470 to nt 1446). Reverse transcription and polymerase chain reactions (PCR) were carried out according to the instruction manual of the SMART RACE cDNA Amplification Kit. The 5'- and 3'-RACE products were gel-purified using the S.N.A.P.™ Gel Purification Kit (Invitrogen, USA), TA-cloned into pCRII-TOPO (Invitrogen, USA) and seeded on kanamycin IPTG plates. Miniprep were obtained from colonies grown in 5 ml LB-kanamycin broth and the clones were sequenced on the ABI-3730 Genetic Analyzer using the Big Dye terminator chemistry (Applied Biosystems, USA).

### Molecular cloning of full-length cDNA

Total RNA from PMA-stimulated PBMCs was reverse transcribed using Improm II (Promega, USA). The full-length cDNA was then generated by long distance PCR using Platinum Taq DNA polymerase High Fidelity (Invitrogen, USA) with primers designed from the distal ends of both 5'- and 3'-RACE products : 5'-GTCGCCAGTAAATCCCAAGA-3' (sense, within the 5'-UTR) and 5'-GCACCTCAATCTCCACCACT-3' (antisense, 3'UTR). The procedures recommended by the manufacturer were followed, with these cycling parameters : 5 min at 94°C, then 35 cycles including (*i*) 30 s at 94°C, (*ii*) 30 s at 58°C and (*iii*) 3 min 30 s at 68°C, followed by a final extension at 68°C for 5 min. Resulting PCR products were then processed for sequencing as aforementioned for the RACE products. The CD11a cDNA sequence was deduced from sequences obtained from nine independent clones. Sequence data have been deposited at GenBank under accession No. AY773018 and AY773019.

### Bioinformatics

Primers design was performed with Netprimer [18] and Primer 3 [19]. Nucleotidic sequence and similarity analyses were carried out using respectively Chromas v.2.21 [20] and BLAST programs [21]. Alignment of amino acids sequences were drawn by GeneDoc v.2.6.002 [22] following the BLOSUM62 matrix. SignalP v.2.0.b2 [23] and NetNGlyc v.1.0 [24] provided peptide signal and N-glycosylation sites prediction, respectively.

## Results & discussion

### Characterisation of CaCD11a-encoding cDNA and deduced aa sequence

**Figure 1:**
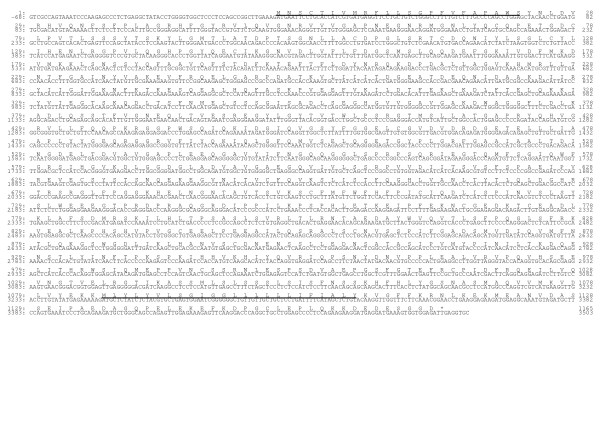
**The nucleotide and deduced amino acid sequences of *Capra hircus *CD11a cDNA. **The putative leader peptide and transmembrane region are underlined. Nine independent clones were sequenced in both directions. Sequence data have been deposited at GenBank under accession No. AY773018 and AY773019 (shown here), respectively without and with Gln-743 (#).

**Figure 2:**
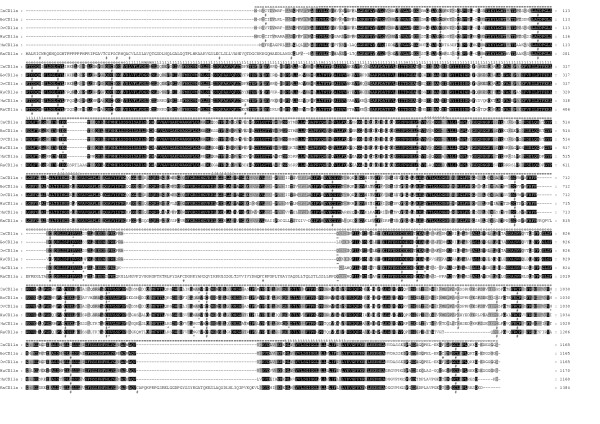
**Comparison of the caprine (Ca-), bovine (Bo-), human (Hu-), murine (Mu-) and rat (Ra-) α subunits amino acid sequences. **The letters in the top row identify the constitutive blocks: putative signal peptide (s), extracellular domain (e), transmembrane region (t), cytoplasmic tail (c), I-domain (i), MIDAS motif (m) and divalent cation binding motifs (d). Black, dark grey and light grey columns represent identity among 6, 5 and 4 species, respectively. Cysteine residues (#) and potential N-glycosylation sites (*) are marked at the bottom of the sequences. The important Glu-332 residue (+) and the Gln-743 residue which is absent in the smaller allele (=) are identified.

Two alleles have been identified for the CaCD11a cDNA. The sequence contains ~4200 bp with an ORF of 3498 [Genbank: AY773019] or 3495 bp [Genbank: AY773018] depending on the allele that codes for 1165 or 1164 aa followed by ~600 bp in the 3'-UTR (Fig. 1). The mature CaCD11a contains a 23-aa putative leader peptide, an extracellular domain of 1061 or 1062 residues (24-1084/1085), a single hydrophobic transmembrane region of 24 residues (1085/1086–1108/1109) and a cytoplasmic tail of 57 residues (Fig. 1). Nine N-linked putative glycosylation sites (Asn-X-Thr/Ser) are present in the extracellular domain (Fig. 2). The mature protein contains 19 cysteine residues among which one is located into the cytoplasmic tail (Fig. 2). The extracellular domain also contains an inserted (I) domain of 172 amino acids (residues 153–324) quite similar to those found in all the leukocyte integrin α subunits sequenced to date and located between the β sheets 2 and 3 of a seven bladed β-propeller region [25]. The I-domain is homologous with repeated domains found in von Willebrand factor and cartilage matrix protein [1] and can be expressed as an isolated domain. Its three-dimensional structure consists of a five-stranded parallel β-sheet core surrounded on both faces by α-helices, with a short antiparallel strand occurring on one edge of this sheet [26]. The I-domain contains a metal ion-dependent adhesion site (MIDAS) [27] (residues 159–163, 228, 261) (Fig. 2) and an I-domain allosteric site (IDAS) that plays a functional role in ICAM-1 binding [28-30]. Three repeats with a divalent cation binding motif are found at amino acid residues 465–473, 527–535 and 587–595 (Fig. 2). All the conserved cysteines and all but one N-glycosylation sites are found outside the I region and divalent cation binding motifs (Fig. 2), consistent with the hypothesis that these regions may undergo conformational changes important in ligand binding [31,32].

### General comparison among species

Overall, the general organization of caprine, bovine [33], ovine [34], human [32], murine [35] and rat [GenBank: NW_047562] CD11a proteins is quite similar (Fig. 2). Comparison between mature CaCD11a sequence and its bovine, ovine, human, murine and rat counterparts shows overall 94%, 98%, 77%, 68% and 55% identity, respectively, with the highest identity for the MIDAS, the cation binding motifs and the transmembrane region and the lowest identity for the cytoplasmic tail (Table 1). The high conservation of the MIDAS and the putative cation binding motifs is consistent with an involvement of these regions in the functional activity of LFA-1 α subunit, as suggested by the requirement of Mg^2+ ^and Ca^2+ ^for CD11a/CD18-dependent cellular interactions [31] or binding to purified ICAM-1 [36,37]. The transmembrane region shows also a high degree of conservation that could be explained by (i) physicochemical, and (ii) functional constraints. Indeed, (i) residues lying in the membrane have to possess rather hydrophobic character to allow liposolubility, which is confirmed by the presence of many leucine residues (figure 2) and (ii) bi-directional integrin signalling (inside-out and outside-in) is accomplished by transmission of information across the plasma membrane [38]. By contrast, the low conservation of the cytoplasmic tail suggests that it is not required to guarantee adequate functioning of LFA-1. This is in agreement with the observation that truncation of the LFA-1 α subunit cytoplasmic domain has no effect on binding to ICAM-1, whereas binding is markedly diminished by β subunit cytoplasmic domain truncation [39]. Residue Glu-332 that is located in the linker following the I-domain and that is known to be critical for communication to the β_2 _I-like domain, rolling, integrin extension and activation by Mn^2+ ^of firm adhesion [8] is strictly conserved.

**Table 1:** Between-species percent identities of CD11a constitutive blocks. Ca, Bo, Ov, Hu, Mu and Ra: caprine, bovine, ovine, human, murine and rat CD11a, respectively; MIDAS: metal-ion dependent adhesion site.

Block	Ca vs. Bo (%)	Ca vs. Ov (%)	Ca vs. Hu (%)	Ca vs. Mu (%)	Ca vs. Ra (%)
Overall	94	98	77	68	55
Putative signal peptide	86	95	60	41	4
Extracellular domain	94	98	78	69	60
Transmembrane region	95	100	95	79	87
Cytoplasmic tail	92	94	58	50	47
I-domain	95	99	86	72	75
MIDAS	100	100	100	85	85
Putative cation binding motif 1	100	100	88	66	55
Putative cation binding motif 2	100	100	77	88	88
Putative cation binding motif 3	100	100	88	77	88

Every cysteine residue in the caprine extracellular portion of mature CD11a is present at the same location in bovine, ovine, human, murine and rat CD11a, which is consistent with a role in maintaining the global structure of the protein whereas two cysteine residues (positions 1009 and 1048) are absent from caprine CD11a and therefore do not seem to be indispensable. The mouse version distinguishes by an additional cysteine residue at position 199 (mouse numbering) within the extracellular portion. Of nine potential Asn-glycosylation sites in caprine CD11a, the ones present at amino acids 185, 667, 723 and 859 are strictly conserved, one is only absent from murine and rat CD11a (residue 894), without predictable consequences on a functional point of view.

Interestingly, as in sheep [34] and human [GenBank: NM_002209 and AY892236], an allelic variant with a triplet insertion resulting in an additional Glu744 in the extracellular domain was consistently identified, which suggests an allelic polymorphism that might be biologically relevant. Studies of genomic sequences will permit to know if this addition represents two alleles or not.

Finally, one has to note that the lowest between-species percent identities are observed with the rat CD11a sequence which has been derived from an annotated genomic sequence. Cloning and characterisation of rat CD11a from rat PBMCs would probably give a higher identity.

## Conclusion

This study reports for the first time the isolation and sequencing of the caprine LFA-1 α subunit (CD11a) cDNA, and demonstrates that, despite some focal differences, it shares all the main characteristics of its known mammalian homologues. Along with the caprine CD18-encoding cDNA which is now available [17], the sequence data provided here will allow the successful expression of caprine LFA-1 *in vitro *as a tool to examine the specificities of inflammation in the caprine species.

## Competing interests

The author(s) declare that they have no competing interests.

## Authors' contributions

TF carried out cloning and sequencing, participated in the sequence alignment and to the draft of the manuscript. LZ participated in the sequence comparison and to the draft of the manuscript. EB participated in the design of the study. DD conceived the study, and participated in its design and coordination and helped to draft the manuscript. All authors read and approved the final manuscript.
